# Risk factors and outcomes for airway failure versus non-airway failure in the intensive care unit: a multicenter observational study of 1514 extubation procedures

**DOI:** 10.1186/s13054-018-2150-6

**Published:** 2018-09-23

**Authors:** Samir Jaber, Hervé Quintard, Raphael Cinotti, Karim Asehnoune, Jean-Michel Arnal, Christophe Guitton, Catherine Paugam-Burtz, Paer Abback, Armand Mekontso Dessap, Karim Lakhal, Sigismond Lasocki, Gaetan Plantefeve, Bernard Claud, Julien Pottecher, Philippe Corne, Carole Ichai, Zied Hajjej, Nicolas Molinari, Gerald Chanques, Laurent Papazian, Elie Azoulay, Audrey De Jong

**Affiliations:** 1 PhyMedExp, University of Montpellier, Anesthesiology and Intensive Care; Anesthesia and Critical Care Department B, Saint Eloi Teaching Hospital, Centre Hospitalier Universitaire Montpellier, 34295 Montpellier, cedex 5 France; 2 Université Cote d’Azur, CNRS U7275, CHU de Nice, Service réanimation polyvalente et U 7275, IPMC, Nice, France; 3 Intensive Care & Anesthesiology Department, University of Nantes, Hotel-Dieu Hospital, Nantes, France; 4 Intensive Care Department, Sainte Musse Hospital, Toulon, France; 5 Medical Intensive Care Unit, Hôtel-Dieu Teaching Hospital, Nantes, France; 6 Intensive Care & Anesthesiology Department, Univ Paris Diderot, Sorbonne Paris Cité, AP-HP, Hôpital Beaujon, F-75018 Paris, France; 7 Service de Réanimation Médicale, DHU A-TVB, Hôpitaux Universitaires Henri Mondor, Assistance Publique-Hôpitaux de Paris, Groupe de Recherche Clinique CARMAS, Faculté de Médecine de Créteil, Université Paris Est Créteil, 94010 Créteil Cedex, France; 8 Intensive Care & Anesthesiology Department, University of Nantes, Laennec Nord Hospital, Nantes, France; 9 Département Anesthésie Réanimation, CHU Angers, 49933 Angers, Cedex 9 France; 10 Medical-Surgical Intensive Care Unit, General Hospital Centre, Argenteuil, France; 11 Medical-Surgical Intensive Care Unit, General Hospital Centre, Le Puy-en-Velay, France; 12 Hôpitaux Universitaires de Strasbourg, Pôle Anesthésie Réanimation Chirurgicale SAMU, Hôpital de Hautepierre, Service d’Anesthésie-Réanimation Chirurgicale, Université de Strasbourg, Fédération de Médecine Translationnelle de Strasbourg (FMTS), Faculté de Médecine, Institut de Physiologie, Equipe d’Accueil EA3072 “Mitochondrie, stress oxydant et protection musculaire”, Strasbourg, France; 13 Medical Intensive Care Unit, Montpellier University Hospital, Montpellier, France; 14 Anesthesiology and Intensive Care; Anesthesia and Critical Care Department B, Saint Eloi Teaching Hospital, Centre Hospitalier Universitaire Montpellier, 34295 Montpellier, cedex 5 France; 15 IMAG, CNRS, Univ Montpellier, CHU Montpellier, Montpellier, France; 16 APHM, URMITE UMR CNRS 7278, Hôpital Nord, Réanimation des Détresses Respiratoires et Infections Sévères, Aix-Marseille Univ, Marseille, France; 17 Medical Intensive Care Unit, University of Paris-Diderot, Saint Louis Hospital, Paris, France

**Keywords:** Airway, Extubation, Non-airway, weaning

## Abstract

**Background:**

Patients liberated from invasive mechanical ventilation are at risk of extubation failure, including inability to breathe without a tracheal tube (airway failure) or without mechanical ventilation (non-airway failure). We sought to identify respective risk factors for airway failure and non-airway failure following extubation.

**Methods:**

The primary endpoint of this prospective, observational, multicenter study in 26 intensive care units was extubation failure, defined as need for reintubation within 48 h following extubation. A multinomial logistic regression model was used to identify risk factors for airway failure and non-airway failure.

**Results:**

Between 1 December 2013 and 1 May 2015, 1514 patients undergoing extubation were enrolled. The extubation-failure rate was 10.4% (157/1514), including 70/157 (45%) airway failures, 78/157 (50%) non-airway failures, and 9/157 (5%) mixed airway and non-airway failures. By multivariable analysis, risk factors for extubation failure were either common to airway failure and non-airway failure: intubation for coma (OR 4.979 (2.797–8.864), *P* < 0.0001 and OR 2.067 (1.217–3.510), *P* = 0.003, respectively, intubation for acute respiratory failure (OR 3.395 (1.877–6.138), *P* < 0.0001 and OR 2.067 (1.217–3.510), *P* = 0.007, respectively, absence of strong cough (OR 1.876 (1.047–3.362), *P* = 0.03 and OR 3.240 (1.786–5.879), *P* = 0.0001, respectively, or specific to each specific mechanism: female gender (OR 2.024 (1.187–3.450), *P* = 0.01), length of ventilation > 8 days (OR 1.956 (1.087–3.518), *P* = 0.025), copious secretions (OR 4.066 (2.268–7.292), *P* < 0.0001) were specific to airway failure, whereas non-obese status (OR 2.153 (1.052–4.408), *P* = 0.036) and sequential organ failure assessment (SOFA) score ≥ 8 (OR 1.848 (1.100–3.105), *P* = 0.02) were specific to non-airway failure. Both airway failure and non-airway failure were associated with ICU mortality (20% and 22%, respectively, as compared to 6% in patients with extubation success, *P* < 0.0001).

**Conclusions:**

Specific risk factors have been identified, allowing us to distinguish between risk of airway failure and non-airway failure. The two conditions will be managed differently, both for prevention and curative strategies.

**Trial registration:**

**ClinicalTrials.gov**, NCT 02450669. Registered on 21 May 2015.

**Electronic supplementary material:**

The online version of this article (10.1186/s13054-018-2150-6) contains supplementary material, which is available to authorized users.

## Background

Mechanical ventilation is a life-saving intervention [1]. In the intensive care unit (ICU), the timing of liberation from invasive mechanical ventilation is an important issue for clinicians caring for critically ill intubated patients receiving mechanical ventilation, and differs from the extubation procedure after elective surgery [1]. The benefit-risk ratio for extubation has to be assessed on a daily basis. If the patient remains intubated too long, complications associated with prolonged mechanical ventilation may appear [2]. On the other hand, if the patient is extubated too early, reintubation is associated with higher mortality and long-term disability [3–5]. Extubation failure is defined as the need for reintubation within 24–72 h [4–8] or up to 7 days [9].

Causes of extubation failure include upper airway obstruction (stridor mainly related to laryngeal edema), lower airway obstruction (aspiration or excessive respiratory secretions), congestive heart failure, respiratory failure, or encephalopathy (decreased consciousness leading to hypoventilation) [10]. After resolution of illness, mechanically ventilated patients are liberated from the ventilator, a process termed “weaning” [8]. Weaning and extubation, though following each other in clinical practice, are two separate processes that pose distinct problems. Extubation failure can be due to “airway failure” and/or “non-airway failure” which also refers to “weaning failure” [5, 10]. Airway failure, defined as the inability to breathe without an endotracheal tube, differs from weaning failure also assimilated to non-airway failure, defined as the inability to breathe without invasive mechanical ventilation [3].

It will be of interest to distinguish between airway failure and non-airway failure/weaning failure because the two conditions will be managed differently, both for prevention and curative strategies.

Several methods for anticipating/managing non-airway failure have been explored, including spontaneous breathing trials (SBT) [11, 12], careful cardiac assessment using brain natriuretic peptide [13] or cardiac ultrasound during SBT [14, 15]. Ultrasound is used to evaluate the heart, diaphragm, pleura and lungs during the weaning process [16–19]. Regarding prevention of airway failure, the cuff-leak test is one of the tools developed for identifying a cause related to upper-airway failure associated with laryngeal edema: post-extubation stridor [20, 21]. Cough expiratory peak-flow and evaluation of the amount of secretions have been proposed as tools to identify patients at risk of developing lower-airway failure [22].

To date, only one single-center retrospective study published in 1998 [10] including 74 medical ICU patients who required reintubation has reported the respective incidences of airway failure (31%) and non-airway failure (69%). To our knowledge, no study has specifically evaluated the risk factors related to airway failure as opposed to non-airway failure, respectively.

We hypothesized that the two mechanisms that lead to extubation failure, namely airway failure and non-airway failure, are also associated with specific determinants of occurrence. We then performed a large multicenter prospective study to identify risk factors for each component of extubation failure.

This work was presented as an abstract at the meeting of the Société de Réanimation de Langue Française (Paris 2017).

## Methods

### Conduct of the study, patient population and inclusion/exclusion criteria

A prospective, observational, multicenter study was conducted in 26 ICUs. All consecutive adult patients extubated in participating ICUs were included. Exclusion criteria included age < 18 years, pregnancy, and terminal extubation [23]. Patients who died before extubation and/or with tracheotomy were not eligible. In patients undergoing more than one extubation episode, each extubation procedure was considered. Additional detail on the method for collecting data is provided in Additional file 1.

### Ethics approval

The appropriate Institutional Review Board (*Comité de Protection des personnes Sud-Mediterranée III*) approved the study protocol (code UF: 9242, register: 2013-A01402–43) and, based on the observational design, waived the need for written informed consent. Next of kin were informed of the study, as were patients, as soon as their neurologic status was deemed adequate. Written information was delivered to the patient’s next of kin and to the patient when neurologic recovery was deemed appropriate. The study was registered on ClinicalTtrials.gov (identifier number NCT 02450669).

### Definition of extubation failure, airway failure and non-airway failure

Extubation failure was defined as a need for reintubation within 48 h after extubation [8]. Patients were categorized into airway failure or non-airway failure according to the principal cause determined by the medical ICU team members. To limit the misclassification of each cause of extubation failure, the participating centers were asked to have two persons classify each reintubated patient, to assess the mechanisms of extubation failure. In case of disagreement and/or difficulty in classification, two independent observers (SJ and ADJ) made the final classification.

Extubation failure due to airway failure was defined as an extubation failure because of the inability to breathe without a tracheal tube, according to previously published definitions by Epstein et al. [10]. Following the Epstein et al. [10] definition, included in this category were upper-airway obstruction and lower-airway obstruction due to aspiration or excessive respiratory secretions (witnessed aspiration or inability to maintain airway patency because of respiratory secretions, defined as the need for repeated naso-tracheal aspiration or the development of atelectasis during the post-extubation period, because of ineffective cough or inability to expectorate) [10].

Extubation failure due to non-airway failure was defined as an extubation failure related to the inability to breathe without invasive mechanical ventilation, according to previously published definitions by Epstein et al. [10]. Following the Epstein et al. [10] definition, congestive heart failure, respiratory failure (lung disease) and hypoventilation were included in this category [10].

Extubation failure due to mixed airway and non-airway failures was defined when a main mechanism (i.e. airway failure or non-airway failure) of reintubation could not be defined (cases of “uncertainty”), because both airway failure and non-airway failure could explain the extubation failure. Figure S1 in Additional file 1 summarizes the definitions of airway failure, non-airway failure and mixed airway and non-airway failures.

### Data handling

The primary outcome was airway failure. The secondary outcomes were non-airway failure, mixed airway and non-airway failures, the rate of difficult intubation in the case of extubation failure, late reintubation (between 2 days and 7 days), the reintubation delays, the use and the length of mechanical or non-invasive ventilation, the need for vasopressors or dialysis after extubation, the occurrence of hospital-acquired infections (nosocomial pneumonia, catheter infections, bacteremia, urinary infections) and mortality at day 28.

### Statistics

The number of subjects to be included in the study was calculated to obtain composite criteria for airway failure. Considering sensitivity of 90% ± 7% based upon a 7% incidence of airway failure [3, 10, 20], it was estimated that 1015 extubation procedures would be required. Taking missing data into account, we decided to include 1500 extubation procedures to develop the model. This sample size also enabled us to obtain composite criteria for non-airway failure (with an estimated incidence at 5%) [3, 10, 20].

Quantitative variables were expressed as means (standard deviation) or medians (interquartiles 25–75%) and compared using the Student *t* test or the Wilcoxon test as appropriate (Gaussian or non-Gaussian variables). Qualitative variables were compared using the chi-squared test or the Fisher test as appropriate.

Patients with mixed airway failures and non-airway failures were excluded from the first analysis. As the dependent variable (extubation failure) consists of three non-ordinal categories, airway failure, non-airway failure and extubation success and were analyzed by multinomial logistic regression [24]. The multinomial logistic regression allows simultaneous comparison of “airway failure” and “non-airway failure” with “extubation success”. A multivariate multinomial logistic model was established. Interactions between variables were tested. All variables with *P* values < 0.20 in the univariate multinomial logistic regression analysis were entered into the model and a backward procedure was used to select the final model, keeping only significant variables with *P* values < 0.05. Odds ratios (ORs) with 95% confidence intervals (CIs) for response were calculated using “Extubation success” as the reference category. The effect of center was assessed by entering this variable in a random effects model as a fixed and random effect [25].

In a second analysis (sensitivity analysis), patients with mixed airway and non-airway failures were included in both the airway failure and non-airway failure groups. In a third analysis (sensitivity analysis), only the first extubation procedure for each patient was included. In a fourth analysis (sensitivity analysis), excessive respiratory secretions were included in the non-airway failure group instead of the airway failure group. In the case of missing values (considered as missing completely at random (MCAR)), no method of replacement was used. A complete case analysis was done (listwise deletion).

A *P* value ≤ 0.05 was considered statistically significant. The statistical analysis was performed by the medical statistical department of the Montpellier University Hospital with the help of statistical software (SAS, version 9.3; SAS Institute; Cary, NC, USA and R, version 2.14.1).

## Results

**Fig. 1:**
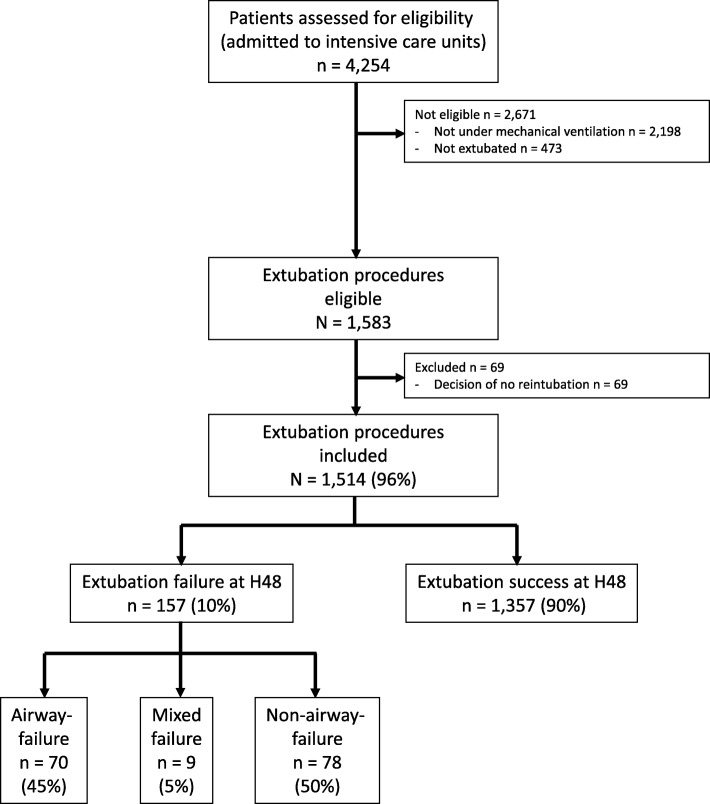
Flow chart for the study. From December 2013 to May 2015, 1514 extubation procedures were studied in 1453 patients from 26 centers. All extubation procedures were included: 61 patients (4.0%) were intubated twice. The median (interquartile range, IQR) number of intubation procedures included per center was 27 (11–72). The incidence of extubation failure (H48 means during the 48 hours following extubation) was 10.4% (157 of 1514 intubation procedures), with “airway”-failure, non-airway failure and mixed airway and non-airway failure incidences, respectively, of 4.6% (70 of 1514), 5.2% (78 of 1514) and 0.6% (9 of 1514)

From December 2013 to May 2015, 1514 extubation procedures were studied in 1453 patients from 26 centers. All the extubation procedures were included: 61 patients (4.0%) were intubated twice. The median (interquartile range, IQR) number of procedures enrolled in each center was 27 (11–72). The flow chart for the study is shown in Fig. 1. The incidence of extubation failure was 10.4% (157 of 1514 intubation-procedures), with airway failure, non-airway failure and mixed airway and non-airway failures incidences of 4.6% (70 of 1514), 5.2% (78 of 1514) and 0.6% (9 of 1514), respectively. Among the 157 extubation procedures, 26 (17%) were misclassified or not classified and needed final classification by the two independent observers.

Table 1 and Additional file 1: Table S1 summarizes information on patient and intubation characteristics, the parameters before extubation and the SBTs performed, and Table S2 (Additional file 1) provides information on the usual functional parameters predicting extubation failure, according to airway failure and non-airway failure, compared to extubation success. The main parameters evaluated during and after the extubation procedure are presented in Table 2.

**Table 1:** Patient and intubation characteristics, parameters before extubation and spontaneous breathing trial according to airway failure, non-airway failure and extubation success with corresponding crude odds ratios determined using multinomial logistic regression

Characteristic	Extubation success (*n* = 1357)	Airway failure (*n* = 70)				Non-airway failure (*n* = 78)			
			OR	95% CI	*P* value		OR	95% CI	*P* value
Age, years	61 (49–71)	61 (51–71)	1.002	0.987–1.017	0.79	65 (51–72)	1.009	0.994–1.023	0.24
Female sex	490/1352 (36)	36/69 (52)	1.919	1.181–3.118	0.009	30 (38)	1.099	0.688–1.758	0.69
SAPS2	49 (36–62)	48 (40–56)	1.010	0.996–1.024	0.18	48 (37–62)	1.019	1.006–1.032	0.004
SOFA score before extubation	2 (0–4)	2 (1–3)	0.954	0.876–1.039	0.28	3 (1–5)	1.054	1.009–1.101	0.02
SOFA score ≥ 8 before extubation	107 (8)	3 (4)	0.523	0.162–1.691	0.28	15 (19)	2.781	1.532–5.051	0.0008
Weight, kg	75 (63–85)	70 (59–87)	0.990	0.976–1.004	0.18	70 (61–80)	0.982	0.968–0.996	0.01
Height, cm	170 (163–175)	166 (160–174)	0.962	0.937–0.988	0.004	168 (160–175)	0.982	0.958–1.007	0.16
Body mass index (kg/m^2^)	25.5 (22.5–29.4)	26.6 (21.5–28.5)	1.000	0.962–1.039	1.00	24.2 (21.1–27.8)	0.958	0.916–1.002	0.06
Body mass index < 30 kg/m^2^	278 (20)	53 (76)	1.131	0.608–2.105	0.70	64 (82)	1.776	0.900–3.502	0.10
Medical type of admission	589 (43)	39 (56)	1.600	0.986–2.595	0.06	39 (50)	1.272	0.805–2.008	0.30
Smoking	349 (26)	13 (19)	0.659	0.356–1.218	0.18	16 (21)	0.745	0.425–1.309	0.31
COPD	173 (13)	10 (14)	1.141	0.573–2.270	0.71	9 (12)	0.893	0.438–1.821	0.75
Alcoholism	295 (22)	14 (20)	0.900	0.494–1.639	0.73	19 (24)	1.159	0.680–1.975	0.59
Cirrhosis	159 (12)	7 (10)	0.837	0.377–1.860	0.66	8 (10)	0.861	0.407–1.823	0.70
Chronic renal failure	168 (12)	5 (7)	0.544	0.216–1.371	0.20	7 (9)	0.698	0.316–1.542	0.37
Reason for ICU admission									
Acute respiratory failure	286 (21)	21 (30)	1.605	0.947–2.720	0.08	21 (27)	1.380	0.823–2.314	0.22
Trauma	103 (8)	9 (13)	1.796	0.867–3.720	0.11	2 (3)	0.320	0.078–1.323	0.12
Post-operative	488 (36)	11 (16)	0.332	0.173–0.638	0.0009	20 (26)	0.614	0.365–1.033	0.07
Cardiac arrest	42 (3)	1 (1)	0.454	0.062–3.346	0.44	7 (9)	3.087	1.339–7.115	0.008
Neurologic failure	356 (26)	38 (54)	2.626	1.604–4.299	0.001	28 (36)	1.368	0.815–2.293	0.24
Shock	242 (18)	13 (19)	1.051	0.566–1.950	0.88	14 (18)	1.008	0.556–1.827	0.98
Ascetic decompensation	24 (2)	1 (1)	0.805	0.107–6.037	0.83	0 (0)	–	–	0.98
Acute renal failure	31 (2)	2 (3)	1.258	0.295–5.367	0.76	2 (3)	1.126	0.264–4.791	0.87
Others	115 (8)	3 (4)	0.484	0.150–1.562	0.22	7 (9)	1.065	0.479–2.369	0.88
Reason for intubation									
Acute respiratory failure	298 (22)	26 (37)	2.100	1.272–3.468	0.004	24 (31)	1.579	0.960–2.598	0.07
Shock	146 (11)	10 (14)	1.382	0.693–2.759	0.36	6 (8)	0.691	0.295–1.618	0.39
Coma	308 (23)	29 (41)	2.409	1.473–3.941	0.0005	24 (31)	1.514	0.921–2.489	0.10
Cardiac arrest	43 (3)	1 (1)	0.443	0.060–3.264	0.42	8 (10)	3.492	1.582–7.711	0.002
Surgery	451 (33)	9 (13)	0.297	0.146–0.603	0.0008	16 (21)	0.518	0.296–0.909	0.02
Others	135 (10)	3 (4)	0.484	0.150–1.562	0.22	8 (10)	1.065	0.479–2.369	0.88
Length of intubation (days)	2.0 (1.0–6.0)	4.5 (1.0–9.0)	1.029	0.997–1.061	0.07	3.5 (1.0–7.0)	1.038	1.011–1.067	0.007
Length of intubation > 8 days	203 (15)	20 (29)	2.174	1.267–3.729	0.005	14 (18)	1.268	0.695–2.312	0.439
Strong cough strength	546 (40)	20 (29)	0.594	0.350–1.009	0.05	12 (15)	0.270	0.145–0.504	< 0.0001
Copious endotracheal secretions	147 (11)	23 (33)	4.028	2.377–6.825	< 0.0001	6 (8)	0.686	0.293–1.605	0.38

Data are summarized as number of extubation procedures/total number of extubation procedures (%) or median (interquartile range). One patient can have more than one reason for ICU admission or for intubation. All *P* values and ORs result from univariate multinomial logistic regression predicting the two modalities of extubation failure (airway failure versus non-airway failure) according to the characteristics

*OR* odds ratio, *CI* confidence interval, *SAPS2* simplified acute physiologic score, *SOFA* sequential organ failure assessment, *COPD* chronic obstructive respiratory disease

**Table 2:** Parameters during and after extubation according to airway failure, non-airway failure and extubation success with corresponding crude odds ratios determined using multinomial logistic regression

Characteristic	Extubation success (*n* = 1357)	Airway failure (*n* = 70)				Non-airway-failure (*n* = 78)			
			OR	95% CI	*P* value		OR	95% CI	*P* value
Operator performing extubation									
Senior	368/1269 (29)	24/63 (38)	–	–		23/69 (33)	–	–	
Junior	451/1269 (36)	13/63 (21)	0.499	0.253–0.983	0.04	21/69 (30)	0.838	0.462–1.519	0.68
Nurse	450/1269 (35)	26/63 (41)	1.129	0.637–1.999	0.56	25/69 (36)	1.125	0.628–2.015	0.69
Extubation at the end of inspiration	121/1143 (11)	8/58 (14)	1.120	0.471–2.665	0.80	7/66 (11)	1.190	0.469–3.022	0.71
Extubation at the end of expiration	108/1143 (9)	4/58 (7)	0.625	0.206–1.900	0.41	7/66 (11)	1.431	0.556–3.682	0.46
Extubation without preference	914/1143 (80)	46/58 (79)	3.422	0.465–25.197	0.23	52/66 (79)	3.869	0.527–28.414	0.18
Suctioning before extubation	1123 (83)	52 (74)	0.787	0.380–1.629	0.52	63 (81)	1.073	0.504–2.282	0.86
FiO2 set at 100% before extubation	417 (31)	21 (30)	1.294	0.732–2.289	0.38	25 (32)	1.101	0.661–1.831	0.71
Recruitment maneuvers before extubation	127 (9)	5 (7)	0.959	0.373–2.464	0.93	5 (6)	0.665	0.262–1.684	0.39
Accidental extubation	6 (0)	1 (1)	6.541	0.672–63.699	0.11	0 (0)	–	–	0.98
Self-extubation	69 (5)	5 (7)	1.436	0.560–3.681	0.45	7 (9)	1.840	0.816–4.151	0.14
Extubation protocol	441 (32)	14 (20)	0.519	0.286–0.943	0.03	24 (31)	0.923	0.563–1.513	0.75
Patient informed of extubation	1225 (90)	64 (91)	1.149	0.488–2.705	0.75	67 (86)	0.656	0.338–1.273	0.21
Daytime extubation	896 (66)	55 (79)	2.141	1.275–3.593	0.004	58 (74)	1.305	0.746–2.282	0.35
Physiotherapy	672 (50)	46 (66)	1.954	1.179–3.237	0.009	46 (59)	1.465	0.922–2.329	0.11
Before extubation	283/672 (42)	23/46 (50)	0.792	0.341–1.840	0.59	17/46 (37)	1.171	0.383–3.582	0.78
Between extubation and 1 h after	470/672 (70)	31/46 (67)	0.923	0.314–2.712	0.88	33/46 (72)	1.966	0.459–8.413	0.36
More than 1 h after	236/672 (35)	12/46 (26)	0.416	0.177–0.977	0.04	22/46 (48)	2.796	0.817–9.568	0.10
Preventive NIV post extubation	290 (21)	22 (31)	1.757	0.697–4.432	0.23	28 (36)	2.237	0.905–5.527	0.08
Curative NIV post extubation	238 (18)	11 (16)	0.877	0.454–1.694	0.70	16 (21)	1.213	0.688–2.139	0.50
Inhaled corticosteroids post extubation	68 (5)	13 (19)	4.373	2.279–8.392	< 0.0001	6 (8)	1.586	0.665–3.780	0.30
Inhaled epinephrine post extubation	40 (3)	17 (24)	10.519	5.593–19.781	< 0.0001	3 (4)	1.341	0.405–4.438	0.63

Data are summarized as number of extubation procedures/total number of extubation procedures (%) or median (interquartile range). All *P* values and ORs result from a univariate multinomial logistic regression predicting the two modalities of extubation failure (airway failure versus non-airway failure) according to the characteristics

*OR* odds ratio, *CI* confidence interval, *FiO2* fraction of inspired oxygen, *NIV* non-invasive ventilation

**Fig. 2:**
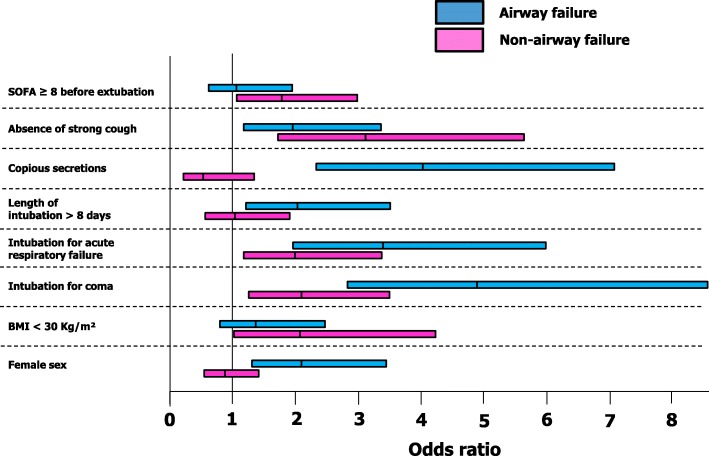
Risk factors in the final model for predicting airway failure, non-airway failure and extubation-failure. BMI, body mass index; SOFA, sequential organ failure assessment. In the final multivariate model constructed with the 1365 extubation procedures and all available data, the main predictors of airway failure were related to patient characteristics and conditions prior to extubation: female gender (OR 2.024 (1.187–3.450), *P* = 0.010), baseline pathology with coma as a reason for intubation (OR 4.979 (2.797–8.864), *P* <£0.0001), acute respiratory failure as a reason for intubation (OR 3.395 (1.877–6.138), *P* < 0.0001), length of ventilation > 8 days (OR 1.956 (1.087–3.518), *P* = 0.025), copious secretions at the time of extubation (OR 4.066 (2.268–7.292), *P* < 0.0001) and absence of strong cough before extubation (OR 1.876 (1.047–3.362), *P* = 0.035). The main predictors of non-airway failure were also related to patient characteristics and conditions prior to extubation: non-obese status (OR 2.153 (1.052–4.408), *P* = 0.036), baseline pathology with coma as a reason for intubation (OR 2.177 (1.301–3.642), *P* = 0.003), acute respiratory failure as a reason for intubation (OR 2.067 (1.217–3.510), *P* = 0.0072), absence of strong cough before extubation (OR 3.240 (1.786–5.879), *P* = 0.0001) and a SOFA score ≥ 8 (OR 1.848 (1.100–3.105), *P* = 0.02)

In the final, multivariate model, the main predictors of airway failure were related to patient characteristics and conditions prior to extubation: female gender (OR 2.024 (1.187–3.450), *P* = 0.010), baseline pathology with coma as a reason for intubation (OR 4.979 (2.797–8.864), *P* < 0.0001), acute respiratory failure as a reason for intubation (OR 3.395 (1.877–6.138), *P* < 0.0001), length of ventilation > 8 days (OR 1.956 (1.087–3.518), *P* = 0.025), copious secretions at the time of extubation (OR 4.066 (2.268–7.292), *P* < 0.0001) and absence of strong cough before extubation (OR 1.876 (1.047–3.362), *P* = 0.035) (Fig. 2). The main predictors of non-airway failure were also related to patient characteristics and conditions prior to extubation: non obese status (OR 2.153 (1.052–4.408), *P* = 0.036), baseline pathology with coma as a reason for intubation (OR 2.177 (1.301–3.642), *P* = 0.003), acute respiratory failure as a reason for intubation (OR 2.067 (1.217–3.510), *P* = 0.0072), absence of strong cough before extubation (OR 3.240 (1.786–5.879), *P* = 0.0001) and sequential organ failure assessment (SOFA) score ≥ 8 (OR 1.848 (1.100–3.105), *P* = 0.02) (Fig. 2).

A center effect was assessed both as a fixed and random effect, but was not significant in the final model. After sensitivity analysis, including mixed airway and non-airway failures both in the airway failure and non-airway failure groups, all but one (length of ventilation > 8 days, *P* = 0.066 for airway failure) of the same risk factors as in the main analysis were encountered. After additional sensitivity analysis, including only the first extubation for each patient, all but one (non-obese status, *P* = 0.054 for non-airway failure) of the same risk factors as in the main analysis were encountered. In a last sensitivity analysis, including excessive respiratory secretions in the non-airway failure group, the same risk factors but one (strong cough in the airway failure, *P* = 0.102) as in the main analysis were encountered. Additional details for sensitivity analysis are provided in Additional file 1.

Tables 3 and 4 present the main outcomes according to airway failure, non-airway failure and extubation success. Reintubation delays were longer in the case of non-airway failure when compared to airway failure (Table 3). ICU and hospital mortality rates, hospital-acquired infection rate, and lengths of stay in the ICU and in hospital were higher in the case of airway failure and non-airway failure (Table 4), as compared to extubation success. Overall, 268 patients (17.7%) were reintubated throughout the ICU stay, including 54 (3.6%) from day 2 to day 7, and 57 (3.8%) between day 7 and ICU discharge.

**Table 3:** Causes and time to reintubation according to airway failure and non-airway failure with corresponding crude odds ratios determined using multinomial logistic regression

Characteristic	Airway failure (*n* = 70)	Non-airway failure (*n* = 78)	*P* value
Reintubation at 48 h	70 (100)	78 (100)	–
Reintubation delay (hours)	10.0 (4.0–24.0)	24.0 (8.0–36.0)	0.004
Cause of reintubation			–
Hypoxia (SpO2 < 90%)	36 (51)	47 (60)	0.28
Tachypnoea > 25/min	30 (43)	48 (62)	0.02
Low arterial pressure (SAP < 80 mmHg)	2 (3)	7 (9)	0.17
Tachycardia > 100/min	17 (24)	30 (38)	0.06
Cardiac arrest	0 (0)	5 (6)	0.06
Agitation	10 (14)	6 (8)	0.20
Coma	23 (33)	12 (15)	0.01
Difficult reintubation	5 (7)	2 (3)	0.26
Stridor	17 (24)	4 (5)	0.0009

Data are summarized as number of extubation procedures/total number of extubation procedures (%) or median (interquartile range)

*SpO2* peripheral oxygen saturation, *SAP* systolic arterial pressure

**Table 4:** Main outcomes according to airway failure, non-airway failure and extubation success with corresponding crude odds ratios determined using multinomial logistic regression

Characteristic	Extubation success (*n* = 1311)	Airway failure (*n* = 65)				Non-airway failure (*n* = 77)				Airway vs non-airway failure
			OR	95% CI	*P* value		OR	95% CI	*P* value	*P* value
		–	–	–	–	–	–	–	–	
Vasopressor use	97 (7)	16 (25)	4.087	2.240–7.454	< 0.0001	19 (25)	4.100	2.347–7.162	< 0.0001	0.99
Dialysis use	54 (4)	6 (9)	2.367	0.979–5.724	0.06	8 (10)	2.701	1.237–5.897	0.01	0.82
Hospital-acquired infections	120 (9)	22 (34)	5.078	2.939–8.775	< 0.0001	30 (39)	6.335	3.862–10.393	< 0.0001	0.53
Pneumonia	63 (5)	16 (25)	6.468	3.485–12.006	< 0.0001	26 (34)	10.099	5.910–17.258	< 0.0001	0.23
Catheter	23 (2)	3 (5)	2.713	0.794–9.275	0.11	3 (4)	2.270	0.666–7.734	0.19	1.00
Bloodstream	63 (5)	5 (8)	1.651	0.640–4.255	0.30	10 (13)	2.957	1.452–6.020	0.003	0.77
Urinary tract	29 (2)	5 (8)	3.684	1.377–9.853	0.009	5 (7)	3.070	1.154–8.166	0.02	1.00
Length of ICU stay	6.0 (2.0–13.0)	17.5 (11.0–30.0)	1.038	1.025–1.051	< 0.0001	16.5 (11.0–26.0)	1.025	1.012–1.039	0.0002	0.32
Length of hospital stay	17.0 (9.0–31.0)	28.5 (18.0–47.0)	1.011	1.005–1.017	0.0005	26.0 (13.0–41.0)	1.009	1.003–1.015	0.0021	0.28
Patient alive at ICU discharge	1237 (94)	52 (80)	0.239	0.125–0.459	< 0.0001	60 (78)	0.211	0.117–0.380	< 0.0001	0.76
Patient alive at hospital discharge	1182 (90)	48 (74)	0.308	0.172–0.552	< 0.0001	53 (69)	0.241	0.144–0.552	< 0.0001	0.51

Data are summarized as number of patients/total number of patients (%) or median (interquartile range). All *P* values and OR result from a univariate multinomial logistic regression predicting the two modalities of extubation failure (airway failure vs non-airway failure) according to the characteristics

*OR* odds ratio, *CI* confidence interval, *ICU* intensive care unit

*In the case of reintubation, several causes of reintubation could be provided for airway failure, non-airway failure or mixed airway and non-airway failures

## Discussion

This study identified respective risk factors for airway failure versus non-airway failure among cases of extubation failure in a large multicenter, prospective cohort of extubated medical-surgical ICU patients. Extubation failure was a frequent event, occurring in 10.4% of cases, with half due to airway failure and half due to non-airway failure. Using multivariate multinomial logistic regression analysis, we identified specific risk factors for airway failure and non-airway failure, respectively.

Anticipating extubation failure is a challenging issue. As observed in the current study for both airway failure and non-airway failure, extubation failure is known to be associated with increased morbidity and mortality [3, 4]. Many studies [26] attempted to identify risk factors for extubation failure in order to prevent it. Nevertheless, the incidence of extubation failure reported in the literature remains quite high, as in the current study, around 10% [3, 27]. Failure in predicting extubation success could be partly explained by the lack of differentiation between airway failure and non-airway failure. The aim of the study defined a priori was therefore to separate airway and non-airway failure, developing a new concept [28], and not to create a score mixing all the extubation failures. Further studies will be needed to develop and validate scores predicting airway and non-airway failure. Airway failure, defined as an inability to breathe without a tracheal tube, is a different entity from non-aiway failure or weaning failure, defined as an inability to breathe without a ventilator that delivers ventilatory support [10]. In order to attempt improvement in predicting extubation failure and associated morbimortality, we sought to separately identify risk factors for airway failure and non-airway failure by splitting extubation failure as a whole into airway failure and non-airway failure. Multinomial logistic regression is a classification method that generalizes logistic regression to multiclass problems (such as extubation failure), i.e. with more than two possible discrete outcomes (i.e. airway failure, non-airway failure, extubation success) [24]. This study showed that certain risk factors were common to both airway failure and non-airway failure (intubation for coma, intubation for acute respiratory failure, absence of strong cough), three risk factors were specific to airway failure (female sex, length of ventilation > 7 days, copious secretions) and two others specific to non-airway failure (non-obese status, SOFA score ≥ 8) (Fig. 2).

To our knowledge, this is the first time that an analysis of failure to be liberated from invasive mechanical ventilation, separating airway failure from non-airway failure, has been performed in a large ICU cohort, including 1514 extubation procedures and 157 extubation failures (Fig. 1). Optimal and individualized patient management prior to extubation may be more efficient in preventing extubation failure if the clinician thought separately in terms of airway failure (intensive physiotherapy in the case of low cough-expiratory flow [29, 30], steroids in patients at high risk of stridor [20], sedation-analgesia optimization [31, 32], preparation of appropriate material if extubation is performed [33, 34]) versus non-airway failure (fluid restriction or diuretics [35, 36], preventive use of non-invasive ventilation (NIV) [37], tracheostomy or delayed extubation in the case of diaphragm dysfunction [18, 38, 39] and optimal treatment of pulmonary infection [40]).

The risk factors found in the present study generally agreed with the risk factors for extubation failure identified in the existing literature [3, 4, 41–44]. The strongest predictors for planned extubation failure in a recent study of Thille et al. [42] were also identified as risk factors for extubation failure in the present study: duration of mechanical ventilation longer than 1 week prior to extubation (length of intubation > 8 days in the present study, a specific risk factor for airway failure), ineffective cough (a risk factor for airway failure and non-airway failure), and severe systolic left ventricular dysfunction (correlated with a SOFA score ≥ 8, a risk factor for non-airway failure). Female sex was also found as a risk factor for post-extubation stridor in previous studies, probably resulting from small airway size and a large endotracheal tube size in relation to laryngeal size [45, 46]. Obesity might be associated with a better prognosis in both acute respiratory distress syndrome [47] and overall for ICU patients [48]. The “obesity paradox” also seems present after extubation, and more accurately in non-airway failure following extubation. Baseline diseases (intubation for coma and/or acute respiratory failure) were both risk factors for airway failure and non-airway failure in the present study, and are consistent with the literature on extubation failure. The prevalence of extubation failure is higher in brain-injured patients, respectively 24% and 23% at 48 h in two recent multicenter studies [44, 49]. Additionally, Frutos-Vivar et al. [43] have shown that pneumonia as the reason for initiating mechanical ventilation was an independent risk factor for extubation failure. As in the current study, copious secretions and agitation were identified as risk factors for extubation failure in previous studies [3, 42].

The study has certain limitations and strengths requiring discussion. First, correct classification into airway failure versus non-airway failure was challenging, while the Epstein definitions were used for classification [10]. To limit the misclassification of each cause of extubation failure, two persons in each participating ICU assessed the main cause of extubation failure and in case of disagreement and/or difficulty in classification, two independent observers made the final classification. Moreover, two sensitivity analysis were performed, including either mixed airway and non-airway failures in both the airway failure and non-airway failure groups, or excess respiratory secretions in the non-airway failure group instead of the airway failure group, Both sensitivity analyses showed similar results than in the main analysis. Second, a weaning test was only performed in 77% of the cohort. Despite the primary interest of a well-conducted SBT, variation in SBT performance and documentation across and within sites has been previously described [50]. Moreover, a weaning test may sometimes seem pointless when dealing with a short duration of mechanical ventilation, as all cases of extubation were included in the present study regardless of the duration of mechanical ventilation, which is also a strength of the study. It is worth noting that, for this reason, physiotherapy was used in half of the cases, because it is not systematically used in the participating units in case of reduced length of mechanical ventilation. Third, this pragmatic non-interventional observational study reflected French ICU practices in “real life”. Some specific risk factors, such as cough strength determined using a peak flow system, rapid shallow breathing index, maximal inspiratory and expiratory pressures or airway occlusion pressure, were not assessed in practice, which is also a strength of this observational study, which sought to identify risk factors among those representing usual care. High-flow nasal cannula therapy was not used at this time in the participating centers. Fourth, we cannot exclude that the observed results in the final multivariate models could be the result of sampling variance [51]. However, our results were consistent after several sensitivity analyses (see Additional file 1). Fifth, a few data were missing for the variables included in the multivariate analysis (*n* = 1368/1514, 9.8%). This small amount of missing data, not for the primary outcome, can be considered as missing completely at random (MCAR), which allowed complete case analysis [52].

## Conclusions

To conclude, this is the first large study to differentiate airway failure and non-airway failure among cases of ICU extubation failure. Specific risk factors have been identified, allowing to distinguish between risk of airway failure and non-airway failure. The two conditions will be managed differently, both for prevention and curative strategies. An individualized strategy separating airway failure from non-airway failure may help clinicians improve patient management before liberation from invasive mechanical ventilation.

## Additional file:

Additional file 1 Additional data are presented: data collection in the methods section, sensitivity analyses in the results section, additional **Figure S1** pointing out the definitions used in the study, and two supplemental **Tables S1** and **S2** providing supplemental patients and spontaneous breathing trials characteristics. (DOCX 107 kb)

## Abbreviations

CI: Confidence intervals
ICU: Intensive care unit
MCAR: Missing completely at random
OR: Odds ratio
SBT: Spontaneous breathing trial
