# Supplementary material for: Risk factors and outcomes for airway failure versus non-airway failure in the intensive care unit: a multicenter observational study of 1514 extubation procedures
Source: Crit Care. 2018 Sep 23;22:236. doi: 10.1186/s13054-018-2150-6 (PMC6151191; doi:10.1186/s13054-018-2150-6)
Supplement: Supplementary file 1 — Additional data are presented: data collection in the methods section, sensitivity analyses in the results section, additional Figure S1 pointing out the definitions used in the study, and two supplemental Tables S1 and S2 providing supplemental patients and spontaneous breathing trials characteristics. (DOCX 107 kb) [file 13054_2018_2150_MOESM1_ESM.docx]

**ADDITIONAL FILE 1**

**Risk factors and outcomes for airway** failure vs non-airway failure in intensive care unit. A multicenter observational **study of 1514 extubation procedures**

**Methods**

**Data collection**

Clinical parameters were prospectively assessed before, during and after extubation procedures. The variables were chosen *a priori* based on a review of the anesthesia and critical care literature. The operator was asked to complete the data collection form before performing the extubation. Then, the variables collected during and after the extubation were recorded by an independent observer (a clinician or a trained research staff).

The following data were assessed before extubation : demographic data (age, sex), weight, height, body mass index (BMI), severity scores (Simplified Acute Physiologic Score (SAPS) II at admission, Sequential Organ Failure Assessment (SOFA) score on the day of the procedure), type of admission (medical vs surgical), co morbidities (alcoholism, smoking, cirrhosis, chronic obstructive pulmonary disease (COPD), chronic renal failure), cause of admission (acute respiratory failure, trauma, post-operative, cardiac arrest, neurologic disorder, shock, ascetic decompensation, acute renal failure, other), cause of intubation (acute respiratory failure, shock, neurologic disorder, cardiac arrest, surgery, others), setting of intubation (prehospital, operative room, ICU, other), first intubation during the ICU stay, the device used for intubation (standard laryngoscope, laryngeal mask, videolaryngoscope, other), nasal or oral intubation, difficulty of intubation (number of attempts, trauma during intubation), Mallampati score, Cormack score, length of intubation, date and hour of extubation (daytime procedure was defined as performed from 8 am to 7pm, otherwise it was on-call procedure). Were also collected before extubation, the type and length of spontaneous breathing trial (SBT), the use of a weaning mode before extubation, the assessment of agitation and response to simple orders (opening mouth, shut the tongue, eye tracking, tight the hand), the cough (spontaneous, provoked by aspirations, no cough, weak cough, moderate cough, strong cough), the respiratory rate, the tidal volume and their ratio during SBT, the perception of the extubation by the patient (feeling able or not to breath without a tube), the performing of a leak test before extubation, the blood results at the end of the SBTs, the suction frequency (per hour, per 2 hours, per 3 hours, more than per 3 hours), abundance of endotracheal secretions, the use of systemic steroids before extubation.

Just before and during the extubation, hypotension (systolic blood pressure < 90mmHg) or vasopressors requirement was assessed, as the information of the patient, the nature and number of the operators, the use of FIO2 set at 100% before extubation, tracheal suctioning before extubation, recruitment maneuver, the time of the extubation (at the end of expiration, inspiration, without preference), and the use of an extubation protocol. Rate of accidental extubation, self extubation and physiotherapy use were also recorded,

Just after extubation, were assessed physiotherapy use, use of NIV, inhaled corticosteroids and epinephrine use, and complications occurring during the hour after extubation: hypoxia (SpO2<90%), upper or lower airway obstruction, tachypnea (>25/min), low blood pressure (Systolic Arterial Pressure (SAP) < 80mmHg), acute pulmonary edema, cardiac arrest, agitation, and coma.

At 48 hours post extubation, the occurrence of reintubation was assessed**.** The causes of reintubation (hypoxia (SpO2<90%), tachypnoea (>25/min), low blood pressure (SBP < 80 mmHg), tachycardia (heart rate > 100/min), cardiac arrest, agitation, coma), and time from extubation to reintubation were recorded. The difficulty of reintubation and stridor occurrence were assessed.

Finally, upon ICU discharge, mortality, the length of the ICU stay, the occurrence of late reintubation (> 48h), the use and the length of invasive or non-invasive ventilation, the need for vasopressors or dialysis after extubation and the occurrence of hospital-acquired infections were recorded. Mortality at day 28 following extubation and the length of hospital stay were assessed at hospital discharge.

**Results**

After sensitivity analysis including mixed airway and non-airway failures both in airway-failure and non-airway -failure, the same risk factors but one (length of ventilation > 8 days, *P* = 0.066 for airway-failure) were found than in the main analysis. In the final multivariate model, the main predictors of airway-failure were related to patient: female sex, OR 1.996 (1.177-3.385), *P* = 0.0482, to baseline pathology: coma as reason for intubation, OR 3.612 (2.015-6.474), *P* < 0.0001, acute respiratory failure as reason for intubation, OR 2.981 (1.643-5.409), *P* = 0.0015, length of ventilation > 8 days OR 1.777 (1.012-3.123), *P* = 0.066; and to conditions before extubation: copious secretions OR 4.057 (2.282-7.212), *P* < 0.0001, absence of strong cough, OR 1.880 (1.055-3.353), *P* = 0.024. The main predictors of non-airway-failure were related to patient: non obese status, OR 2.081 (1.008-4.297), *P* = 0.024, to baseline pathology: coma as reason for intubation, OR 2.150 (1.235-3.743), *P* = 0.016, acute respiratory failure as reason for intubation, OR 2.150 (1.235-3.743), *P* = 0.016; and to conditions before extubation: absence of strong cough OR 3.377 (1.784-6.392), *P* = 0.0002, SOFA score ≥ 8, OR 2.912 (1.510-5.616), *P* = 0.0045.

After sensitivity analysis including only first extubation for each patient, the same risk factors but one (non-obese status, *P* = 0.054 for non-airway-failure) were found than in the main analysis. In the final multivariate model, the main predictors of airway-failure were related to patient: female sex, OR 1.964 (1.134-3.400), *P* = 0.0160, to baseline pathology: coma as reason for intubation, OR 4.708 (2.590-8.557), *P* < 0.0001, acute respiratory failure as reason for intubation, OR 3.610 (1.959-6.653), *P* < 0.0001, length of ventilation > 8 days OR 1.924 (1.052-3.518), *P* = 0.034; and to conditions before extubation: copious secretions OR 4.014 (2.207-7.299), *P* < 0.0001, absence of strong cough, OR 1.904 (1.046-3.466), *P* = 0.035. The main predictors of non-airway-failure were related to patient: non obese status, OR 2.027 (0.988-4.162), *P* = 0.054, to baseline pathology: coma as reason for intubation, OR 2.319 (1.374-3.912), *P* = 0.0016, acute respiratory failure as reason for intubation, OR 2.220 (1.298-3.796), *P* = 0.0036; and to conditions before extubation: absence of strong cough OR 3.169 (1.742-5.764), *P* = 0.0002, SOFA score ≥ 8, OR 1.974 (1.168-3.337), *P* = 0.011.

After sensitivity analysis including excessive respiratory secretions in the non-airway-failure group instead of the airway-failure group, the same risk factors but one (strong cough in airway-failure, *P* =0.102) were found than in the main analysis. In the final multivariate model, the main predictors of airway-failure were related to patient: female sex, OR 2.045 (1.165-3.590), *P* = 0.0127, to baseline pathology: coma as reason for intubation, OR 4.654 (2.534-8.546), *P* < 0.0001, acute respiratory failure as reason for intubation, OR 3.306 (1.772-6.168), *P* = 0.0002, length of ventilation > 8 days OR 2.063 (1.116-3.815), *P* = 0.021; and to conditions before extubation: copious secretions OR 3.566 (1.907-6.665), *P* < 0.0001. The main predictors of non-airway-failure were related to patient: non obese status, OR 1.926 (1.000-3.716), *P* = 0.050, to baseline pathology: coma as reason for intubation, OR 2.419 (1.485-3.939), *P* = 0.0004, acute respiratory failure as reason for intubation, OR 2.164 (1.305-3.590), *P* = 0.0028; and to conditions before extubation: absence of strong cough OR 3.422 (1.924-6.085), *P* < 0.0001, SOFA score ≥ 8, OR 1.819 (1.104-2.997), *P* = 0.019.

**Figure S1**

**Definitions of airway-failure, non-airway-failure and mixed airway and non-airway failures**


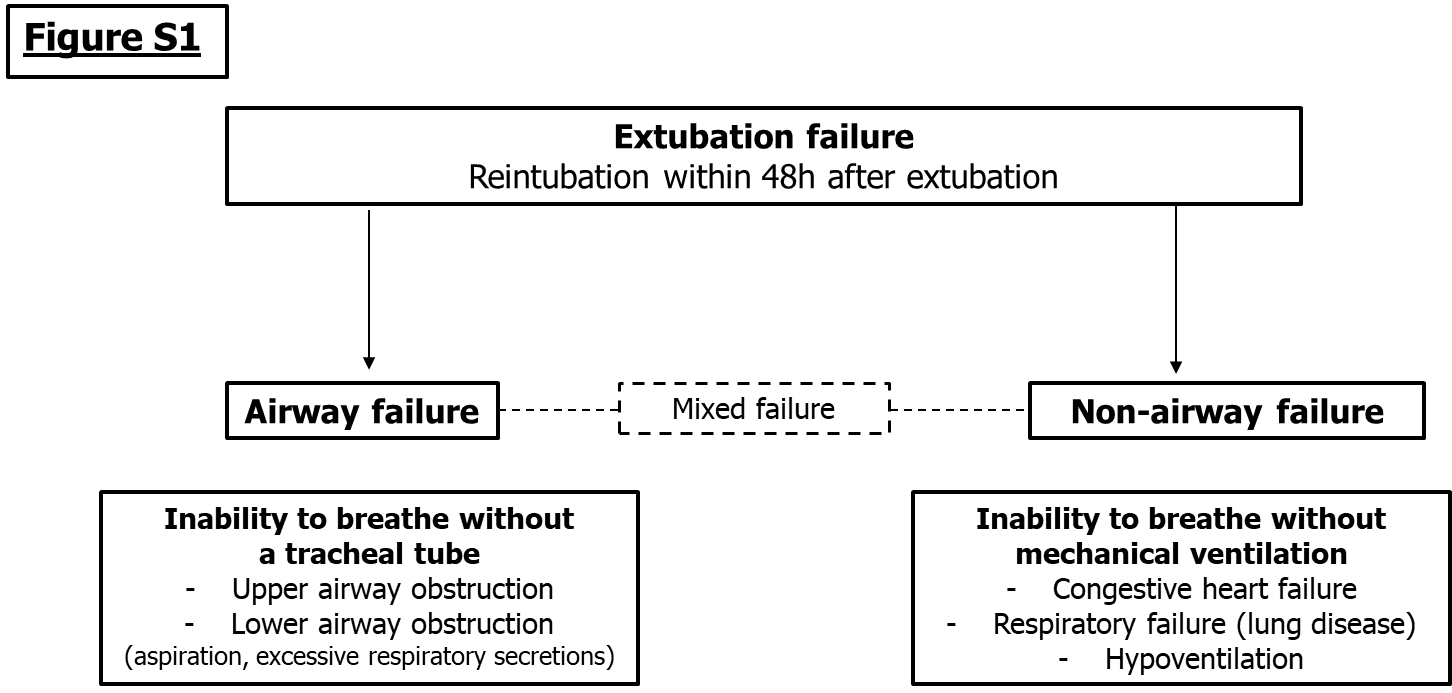


**Table S1. Other patient and intubation characteristics, parameters before extubation and spontaneous breathing trial according to airway-failure, non-airway-failure and extubation success with corresponding crude odds ratios determined using multinomial logistic regression**

| Characteristic | Extubation  success  (n = 1357) | Airway-  failure  (n = 70) |  |  |  | Non-airway-  failure  (n = 78) |  |  |  |
| --- | --- | --- | --- | --- | --- | --- | --- | --- | --- |
|  |  |  | OR | 95% CI | *P* value |  | OR | 95% CI | *P* value |
| Characteristics of participating ICUs |  |  |  |  |  |  |  |  |  |
| Surgical | 478 (35) | 38 (54) | 2.184 | 1.347-3.541 | 0.001 | 32 (41) | 1.279 | 0.804-2.036 | 0.30 |
| Medical | 249 (19) | 11 (16) | 0.830 | 0.430-1.602 | 0.58 | 16 (21) | 1.148 | 0.652-2.024 | 0.63 |
| Mixed medical and surgical | 630 (46) | 21 (30) | 0.495 | 0.293-0.834 | 0.008 | 30 (39) | 0.721 | 0.451-1.152 | 0.17 |
| Presence of an extubation protocol | 441 (32) | 14 (20) | 0·519 | 0·286-0·943 | 0·03 | 24 (31) | 0·923 | 0·563-1·513 | 0·75 |
| Setting of intubation |  |  |  |  |  |  |  |  |  |
| Prehospital | 274 (20) | 17 (24) | 1.268 | 0.723-2.224 | 0.41 | 17 (22) | 1.102 | 0.633-1.916 | 0.73 |
| Operative room | 578 (43) | 18 (26) | 0.529 | 0.263-1.067 | 0.08 | 24 (31) | 0.706 | 0.364-1.368 | 0.30 |
| ICU | 446 (33) | 32 (46) | 1.720 | 1.060-2.790 | 0.03 | 33 (42) | 1.498 | 0.943-2.381 | 0.09 |
| Other | 26 (2) | 2 (3) | 1.713 | 0.382-7.681 | 0.48 | 1 (1) | 0.785 | 0.102-6.032 | 0.82 |
| Difficult intubation | 63 (5) | 5 (7) | 2.352 | 0.902-6.135 | 0.08 | 5 (6) | 1.652 | 0.577-4.725 | 0.35 |
| First intubation | 834 (61) | 49 (70) | 1.463 | 0.868-2.468 | 0.15 | 39 (50) | 0.627 | 0.397-0.991 | 0.05 |
| Trauma during intubation | 20 (1) | 5 (7) | 1.796 | 0.867-3.720 | 0.11 | 1 (1) | 0.320 | 0.078-1.323 | 0.12 |
| Intubation device |  |  |  |  |  |  |  |  |  |
| Standard laryngoscope | 1202 (89) | 62 (89) | 0.999 | 0.470-2.126 | 1.00 | 70 (90) | 1.128 | 0.533-2.389 | 0.75 |
| Laryngeal mask | 5 (0) | 0 (0) | - | - | - | 0 (0) | - | - | - |
| Videolaryngoscope | 26 (2) | 2 (3) | 1.506 | 0.350-6.475 | 0.58 | 1 (1) | 0.665 | 0.089-4.964 | 0.69 |
| Other | 35 (3) | 4 (6) | 2.291 | 0.791-6.635 | 0.13 | 1 (1) | 0.491 | 0.066-3.629 | 0.49 |
| Nasal intubation | 32 (2) | 4 (6) | 2.509 | 0.862-7.340 | 0.09 | 2 (3) | 1.090 | 0.256-4.632 | 0.91 |
| Mallampati score |  |  |  |  |  |  |  |  |  |
| I | 251/550 (46) | 6/17 (35) | - | - | - | 13/24 (54) | - | - | - |
| II | 218/550 (40) | 5/17 (29) | 0.959 | 0.289-3.188 | 0.95 | 8/24 (33) | 0.709 | 0.288-1.741 | 0.45 |
| III | 67/550 (12) | 5/17 (29) | 3.122 | 0.924-10.543 | 0.07 | 2/24 (8) | 0.576 | 0.127-2.617 | 0.48 |
| IV | 14/550 (3) | 1/17 (6) | 2.988 | 0.336-26.549 | 0.33 | 1/24 (4) | 1.379 | 0.168-11.307 | 0.76 |
| Cormack score |  |  |  |  |  |  |  |  |  |
| I | 535/687 (78) | 22/33 (67) | - | - | - | 22/29 (76) | - | - | - |
| II | 83/687 (12) | 5/33 (15) | 1.465 | 0.540-3.975 | 0.45 | 5/29 (17) | 1.465 | 0.540-3.975 | 0.45 |
| III | 46/687 (7) | 5/33 (15) | 2.643 | 0.956-7.306 | 0.07 | 1/29 (3) | 0.529 | 0.070-4.011 | 0.54 |
| IV | 23/687 (3) | 1/33 (3) | 1.057 | 0.137-8189 | 0.96 | 1/29 (3) | 1.057 | 0.137-8189 | 0.96 |
| Corticosteroids before extubation | 119 (9) | 9 (13) | 1.535 | 0.744-3.168 | 0.25 | 9 (12) | 1.357 | 0.661-2.787 | 0.41 |
| Agitation | 247 (18) | 15 (21) | 1.226 | 0.681-2.205 | 0.50 | 10 (13) | 0.661 | 0.355-1.302 | 0.23 |
| Response to simple orders | 1185 (87) | 61 (87) | 0.984 | 0.480-2.017 | 0.96 | 65 (83) | 0.725 | 0.392-1.344 | 0.31 |
| Mouth opening | 880 (65) | 46 (66) | 1.039 | 0.626-1.723 | 0.88 | 46 (59) | 0.779 | 0.490-1.240 | 0.29 |
| Moving tongue | 596 (44) | 35 (50) | 1.277 | 0.790-2.065 | 0.32 | 34 (44) | 0.987 | 0.623-1.563 | 0.95 |
| Eye-tracking | 1072 (79) | 56 (80) | 1.063 | 0.584-1.938 | 0.84 | 59 (76) | 0.826 | 0.484-1.407 | 0.48 |
| Hand strength | 1151 (85) | 61 (87) | 1.213 | 0.593-2.481 | 0.60 | 60 (77) | 0.597 | 0.345-1.031 | 0.06 |
| Patient feeling able to breathe without tube | 618/646 (96) | 23/24 (96) | 0.585 | 0.351-0.975 | 0.04 | 33/35 (94) | 0.877 | 0.553-1.391 | 0.58 |
|  |  |  |  |  |  |  |  |  |  |

Data are summarized as number of extubation procedures/ total number of extubation procedures (%) or median (interquartile range). OR = odds ratio. CI = confidence interval. SAPS2 = simplified acute physiologic score. SOFA = sequential organ failure assessment. COPD = chronic obstructive respiratory disease.

One patient can have more than one reason for ICU admission or for intubation.

All *P* values and ORs result from a univariate multinomial logistic regression predicting the two modalities of extubation failure (airway-failure vs ~~weaning~~ non-airway-failure) according to the characteristics.

**Table S2. Usual functional parameters predicting extubation failure according to airway-failure, non-airway-failure and extubation success with corresponding crude odds ratios determined using multinomial logistic regression**

| Characteristic | Extubation  success  (n = 1357) | Airway-  failure  (n = 70) |  |  |  | Non-airway-  failure  (n = 78) |  |  |  |
| --- | --- | --- | --- | --- | --- | --- | --- | --- | --- |
|  |  |  | OR | 95% CI | *P* value |  | OR | 95% CI | *P* value |
| SBT before extubation realization | 1043 (77) | 57 (81) | 1.389 | 0.736-2.619 | 0.31 | 60 (77) | 0.958 | 0.557-1.648 | 0.88 |
| Length of SBT |  |  |  |  |  |  |  |  |  |
| < 30 min | 368/1043 (35) | 15/57 (26) | - | - |  | 20/60 (33) | - | - |  |
| 30 min to 2h | 472/1043 (45) | 22/57 (39) | 1.144 | 0.585-2.235 | 0.70 | 27/60 (45) | 1.053 | 0.581-1.907 | 0.87 |
| > 2h | 203/1043 (19) | 20/57 (35) | 2.417 | 1.211-4.824 | 0.01 | 14/60 (23) | 1.269 | 0.628-2.566 | 0.51 |
| SBT type |  |  |  |  |  |  |  |  |  |
| T-tube | 448/1043 (43) | 35/57 (61) | 2.109 | 1.302-3.417 | 0.002 | 25/60 (42) | 0.941 | 0.580-1.526 | 0.80 |
| PS-PEEP | 412/1043 (40) | 13/57 (23) | 0.505 | 0.274-0.933 | 0.03 | 24/60 (40) | 0.985 | 0.601-1.614 | 0.95 |
| PS-ZEEP | 164/1043 (16) | 9/57 (16) | 0.999 | 0.503-1.985 | 1.00 | 10/60 (17) | 1.090 | 0.578-2.054 | 0.79 |
| PEEP | 19/1043 (2) | 0/57 (0) | - | - | 0.98 | 1/60 (2) | 0.868 | 0.115-6.554 | 0.89 |
| Peak expiratory flow measured during cough | 3 (0.22) | 1 (1.4) | 6.541 | 0.672-63.699 | 0.11 | 0 (0) | - | - | - |
| Peak expiratory flow | 37.0 (7.0-52.0) | 48.0 (48.0-48.0) | 1.116 | 0.777-1.602 | 0.55 | - | - | - | - |
| Maximal expiratory pressure measured during cough | 3 (0.22) | 0 (0) | - | - | - | 1 (1.3) | 5.861 | 0.603-57.008 | 0.13 |
| Maximal expiratory pressure | 28.0 (15.0-36.0) | - | - | - | - | 28.0 (28.0-28.0) | 1.031 | 0.754-1.410 | 0.85 |
| RR measured during SBT | 683 (50) | 42 (60) | 1.480 | 0.907-2.416 | 0.12 | 35 (45) | 0.803 | 0.508-1.271 | 0.35 |
| Respiratory rate | 20.0 (16.0-24.0) | 20.5 (18.0-25.0) | 1.049 | 1.000-1.101 | 0.05 | 19.5 (17.5-24.0) | 0.994 | 0.937-1.053 | 0.83 |
| TV measured during SBT | 348 (26) | 20 (29) | 1.160 | 0.681-1.976 | 0.59 | 19 (24) | 0.934 | 0.681-1.976 | 0.80 |
| Tidal volume , mL | 450 (400-525) | 440 (353-500) | 0.997 | 0.994-1.001 | 0.16 | 450 (400-550) | 1.000 | 0.996-1.003 | 0.96 |
| Rapid shallow breathing index measured during SBT | 183 (13) | 10 (14) | 1.069 | 0.538-2.126 | 0.85 | 11 (14) | 1.053 | 0.546-2.030 | 0.88 |
| Rapid shallow breathing index, /L | 45.0 (29.4 -69.5) | 39.7 (37.5 -71.0) | 0.994 | 0.963-1.025 | 0.68 | 40.0 (39.4-50.0) | 0.987 | 0.958-1.018 | 0.42 |
| Vital capacity measured during SBT | 4 (0.29) | 0 (0) | - | - | - | 0 (0) | - | - | - |
| Vital capacity, L | 3.0 (2.6 -3.6) | - | - | - | - | - | - | - | - |
| Negative inspiratory force measured during SBT | 44 (3.2) | 2 (2.9) | 0.878 | 0.208-3.696 | 0.86 | 4 (5.1) | 1.613 | 0.564-4.609 | 0.37 |
| Negative inspiratory force | 20.5 (10.0 -31.0) | 10.0 (4.0 -16.0) | 0.903 | 0.756-1.079 | 0.26 | 15.0 (15.0-15.0) | 0.958 | 0.789-1.162 | 0.66 |
| Maximal inspiratory pressure measured during SBT | 0 (0) | 0 (0) | - | - | - | 0 (0) | - | - | - |
| Maximal inspiratory pressure, cm H2O | - | - | - | - | - | - | - | - | - |
| Work of breathing measured during SBT | 0 (0) | 0 (0) | - | - | - | 0 (0) | - | - | - |
| Work of breathing | - | - | - | - | - | - | - | - | - |
| Occlusion pressure measured during SBT | 42 (3.1) | 4 (5.7) | 1.898 | 0.661-5.450 | 0.23 | 2 (2.6) | 0.824 | 0.196-3.468 | 0.79 |
| Occlusion pressure | 2.6 (1.3-3.4) | 2.35 (2.1-2.4) | 0.833 | 0.414-1.673 | 0.61 | 1.75 (1.0-2.5) | 0.626 | 0.193-2.034 | 0.44 |
| PaO2 after SBT measured | 280 (21) | 22 (31) | 1.763 | 1.047-2.970 | 0.03 | 18 (23) | 1.154 | 0.671-1.986 | 0.61 |
| PaO2, mmHg | 92 (76-117) | 84 (76-117) | 0.998 | 0.987-1.009 | 0.67 | 84 (71-114) | 0.997 | 0.984-1.010 | 0.61 |
| PaCO2 after SBT measured | 278 (20) | 23 (33) | 1.899 | 1.134-3.182 | 0.01 | 18 (23) | 1.164 | 0.677-2.004 | 0.58 |
| PaCO2, mmHg | 38 (35-43) | 36 (34-42) | 1.023 | 0.994-1.053 | 0.12 | 37 (36-41) | 1.007 | 0.965-1.052 | 0.74 |
| Leak test performed | 313 (23) | 13 (19) | 0.761 | 0.411-1.408 | 0.38 | 24 (31) | 1.482 | 0.902-2.437 | 0.12 |
| Leaks measured | 79 (5.8) | 3 (4.3) | 0.725 | 0.223-2.355 | 0.59 | 5 (6.4) | 1.108 | 0.435-2.820 | 0.83 |
| Leaks, in mL | 200 (100-310) | 200 (90-375) | 1.000 | 0.996-1.003 | 0.81 | 147 (102-176) | 0.996 | 0.987-1.005 | 0.37 |
| Leaks, in % | 33.9 (18.2-69.8) | 42.6 (25.2-60.0) | 1.000 | 0.951-1.053 | 0.99 | 36.0 (33.3-50.6) | 0.997 | 0.955-1.041 | 0.89 |

Data are summarized as number of extubation procedures/ total number of extubation procedures (%) or median (interquartile range). OR = odds ratio. CI = confidence interval. SBT = Spontaneous breathing trial. PS = pressure support. PEEP = positive end expiratory pressure. CPAP = continuous positive airway pressure. RR = respiratory rate. TV = tidal volume. PaO2 = alveolar oxygen tension. PaCO2 = alveolar carbon dioxide tension. All *P* values and ORs result from a univariate multinomial logistic regression predicting the two modalities of extubation failure (airway-failure vs non-airway-failure) according to the characteristics.
